# Effects of porous tantalum on periprosthetic bone remodeling around metaphyseal filling femoral stem: a multicenter, prospective, randomized controlled study

**DOI:** 10.1038/s41598-022-04936-2

**Published:** 2022-01-18

**Authors:** Goro Motomura, Naohiko Mashima, Hiroshi Imai, Akihiro Sudo, Masahiro Hasegawa, Harumoto Yamada, Mitsuhiro Morita, Naoto Mitsugi, Ryosuke Nakanishi, Yasuharu Nakashima

**Affiliations:** 1 Department of Orthopaedic Surgery, Graduate School of Medical Sciences, Kyushu University, 3-1-1 Maidashi, Higashi-ku, Fukuoka, 812-8582 Japan; 2 Department of Regeneration of Community Medicine, Ehime University Graduate School of Medicine, Shitsukawa, Toon, Ehime, 791-0295 Japan; 3 Department of Orthopaedic Surgery, Graduate School of Medicine, Ehime University, Shitsukawa, Toon, 791-0295 Japan; 4 Department of Orthopaedic Surgery, Mie University Graduate School of Medicine, 2-174 Edobashi, Tsu City, Mie, 514-8507 Japan; 5 Department of Orthopaedic Surgery, Fujita Health University, Toyoake, Aichi 470-1192 Japan; 6 Department of Orthopedic Surgery, Yokohama City University Medical Center, 4-57, Urafune-cho, Minami, Yokohama, Kanagawa 232-0024 Japan; 7 Department of Orthopaedic Surgery, Showa University Fujigaoka Hospital, Showa University School of Medicine, 1-30 Fujigaoka Aobaku, Yokohama, 227-8501 Japan; 8 Department of Orthopedic Surgery, Nisshin Orido Hospital, 110, Nishidamen, Orido-cho, Nisshin, Aichi 470-0115 Japan; 9 Department of Orthopedic Surgery, Osada Hospital, 2-10, Maruyamadai, Minatominami-ku, Yokohama, Kanagawa 233-0013 Japan; 10 Department of Orthopedic Surgery, Shizuoka Medical Center, 762-1, Nagasawa, Shimizu-cho, Sunto District, Shizuoka, 411-8611 Japan

**Keywords:** Randomized controlled trials, Bone

## Abstract

Periprosthetic bone loss due to adaptive bone remodeling is an important unresolved issue in cementless total hip arthroplasty (THA). The use of porous tantalum on the proximal surface of the femoral stem is expected to decrease postoperative bone loss around the prosthesis through early fixation. We conducted a multicenter randomized controlled study to determine if porous tantalum could reduce periprosthetic bone loss after THA. From October 2012 to September 2014, 118 patients (mean age, 61.5 years; 107 females and 11 males) were prospectively enrolled and were randomly allocated at a ratio of 1:1 to either a metaphyseal filling stem with a proximal porous tantalum coating (Trabecular Metal) or a conventional metaphyseal filling stem with fiber mesh coating (VerSys). Patients underwent dual-energy x-ray absorptiometry scans within 1 week after surgery (baseline) and at 6, 12, and 24 months after surgery to assess periprosthetic bone mineral density (BMD) in the 7 Gruen zones. In addition, the Japanese Orthopaedic Association hip score was assessed before surgery and at 6, 12, and 24 months after surgery. In the proximal periprosthetic region (zones 1 and 7), the Trabecular Metal group had significantly smaller reductions in BMD than the VerSys group throughout the study period. In the VerSys group, significant reductions in BMD compared to baseline were seen at each measurement point in all regions, except in zone 6 at 24 months. In the Trabecular Metal group, no significant reductions in BMD relative to baseline were seen in zones 1, 5, or 6 throughout the study period. Both groups demonstrated similar improvement in Japanese Orthopaedic Association hip scores over the study period. This study demonstrated that a proximally coated stem with porous tantalum has superior results over a conventional stem with titanium fiber mesh in terms of periprosthetic bone remodeling.

## Introduction

Total hip arthroplasty (THA) dramatically changes the stress distribution in the femur. Femoral stem placement reduces stress on some areas of the remaining bone^1,2^. Adaptive bone remodeling occurs around the femoral stem, which could result in periprosthetic bone loss^1,2^. Since the greatest difference in stiffness between the stem and femur occurs in the metaphysis, the proximal part of the stem generally tends to experience the most bone loss. Although periprosthetic bone loss secondary to adaptive bone remodeling is not clearly associated with clinical symptoms, it could result in fracture in the long term^3^. In addition, bone loss does raise some concerns for revision surgery, including the risk of fracture during the revision surgery and difficulty of the procedure^1^. Considering that the incidence of revision surgery is projected to increase as the number of primary THA procedures increases^4^, periprosthetic bone loss due to adaptive bone remodeling is an important issue, especially in cementless THA.

For cementless femoral stem fixation, porous coatings are used to promote bone ingrowth to the implant^5^. Proximally coated stems cause less periprosthetic bone loss than fully coated stems^6,7^, indicating that the extent of the porous coating is a factor that affects adaptive bone remodeling. However, it remains unclear whether the types of porous coating affects periprosthetic bone remodeling. Various types of porous coatings, such as porous metals and fiber mesh, have different pore geometrics, mechanical properties, and patterns of bone ingrowth^5^. These may influence adaptive bone remodeling around the femoral stem.

Commercially pure titanium is one of the most commonly used materials for orthopedic implants due to its biocompatibility and biomechanical properties^8^. Titanium fiber mesh, a porous pure titanium, has been successfully used for joint prostheses^9,10^. The structure of titanium fiber mesh provides a high degree of porosity; surface modification of titanium fiber mesh has been shown to promote osteoconductivity^11^. On the other hand, it has some weaknesses such as a high elastic modulus and low shear strength^12^.

Tantalum metal is a highly biocompatible biomaterial applied to joint prostheses^12–14^. Porous tantalum is used on the femoral component surface in THA to enhance fixation properties^12,13^. Porous tantalum has a three-dimensional structure which is similar to that of cancellous bone^12^. Its high volumetric porosity allows a higher rate of bone ingrowth compared to conventional porous coatings^12^. In addition, due to its bone-matched elastic modulus^12^, a reduction in periprosthetic bone loss is theoretically possible. To date, there have been no randomized studies focusing on the effects of porous tantalum on the periprosthetic bone remodeling around the femoral stem.

Compared to titanium fiber mesh, porous tantalum has a higher coefficient of friction^15,16^ suggesting that it leads to better initial fixation. In addition, porous tantalum has been reported to promote osteoblast proliferation and enhance osteogenic potential more than titanium fiber mesh^17^. Increased bone formation in the periprosthetic regions secondary to the osteogenic effect of the porous tantalum and increased load transfer between the implant and bone due to early osseointegration might result in less bone loss. We therefore hypothesized that proximally coated stems with porous tantalum would be more advantageous in reducing periprosthetic bone loss following THA than those coated with titanium fiber mesh. We designed a multicenter randomized controlled study to compare stems with a porous tantalum surface versus a titanium fiber mesh surface stem in terms of periprosthetic bone remodeling. The primary endpoint was change in periprosthetic bone mineral density (BMD) measured with dual-energy x-ray absorptiometry (DEXA) scans over 2-years after THA.

## Materials and Methods

This study (registration number: UMIN000008991, date of first registration: 06/11/2012) was approved by each hospital’s ethics committee. It was conducted in accordance with the Declaration of Helsinki. Written informed consent was obtained from all patients prior to surgery.

**Figure 1:**
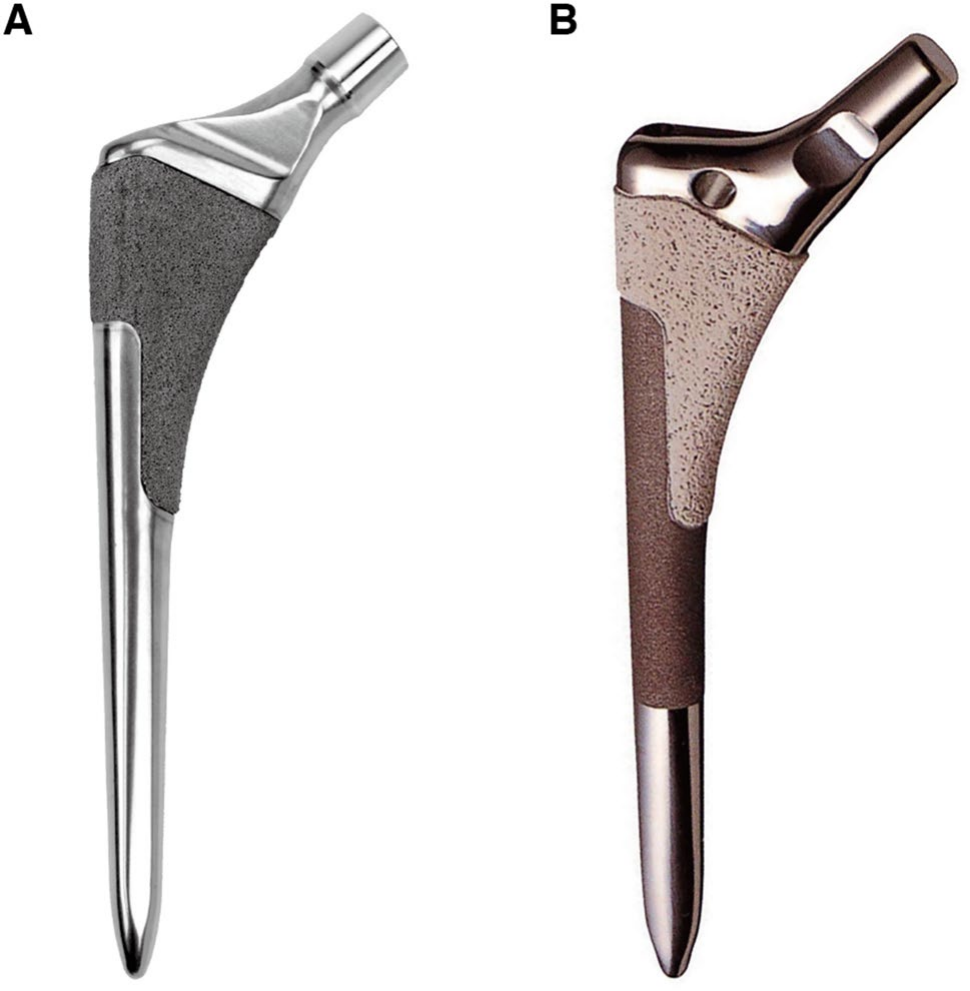
(**A**) The Trabecular Metal Primary Hip Prosthesis with a proximally coating of porous tantalum. (**B**) The VerSys HA/TCP Fiber Metal Taper stem with titanium fiber mesh and an additional proximal coating of calcium phosphate. The surface distal to the proximal coating was corundumized.

Prior to the start of the study, the research sponsor commissioned a third party to prepare an allocation table of 50 cases at 6 participating facilities, for a total of 300 cases. The research physician sent each subject’s enrollment card to the research sponsor after obtaining consent. The research sponsor informed the research physician of the stem assignment according to the allocation table. A total of 118 suitable patients were prospectively enrolled from October 2012 to September 2014. They were randomly allocated at a ratio of 1:1 to either a proximally coated stem with a porous tantalum surface (Trabecular Metal Primary Hip Prosthesis; Zimmer-Biomet, Warsaw, IN) (Fig. 1A) or a conventional stem with a titanium fiber mesh surface (VerSys HA-TCP Fiber Metal Taper Stem; Zimmer-Biomet) (Fig. 1B).

The inclusion criteria included (1) secondary osteoarthritis due to developmental dysplasia of the hip joint and (2) age between 20 and 75 years. Patients were excluded if they (1) were suspected of having poor bone quality, such as being on corticosteroid treatment, (2) had stovepipe canal of the proximal femur, (3) had mental illness that may affect their ability to understand and consent to the study, (4) had undergone ipsilateral or contralateral lower limb surgery such as THA or total knee arthroplasty within 6 months prior to study entry, and (5) were expected to require ipsilateral or contralateral lower limb surgery within 6 months of study entry. If any of the following drugs that might affect bone mineral density had been administered for at least 3 months prior to study entry, the same drug could be continued until the end of the observation period as long as the dose and route of administration remained the same; bisphosphonates, selective estrogen receptor modulators, vitamin D, vitamin K, estrogen, calcium preparations, and ipriflavone.

 The Trabecular Metal Primary Hip Prosthesis is a metaphyseal filling femoral stem, made of a titanium alloy (Ti-6Al-4V) with a proximal coating of porous tantalum called Trabecular Metal™, an ultraporous material with a structure similar to trabecular bone^12^. The VerSys HA/TCP Fiber Metal Taper stem is the same metaphyseal filling femoral component made of titanium alloy (Ti-6Al-4V), but it has a proximal coating of titanium fiber mesh. Hydroxyapatite/tricalcium phosphate (HA/TCP) was applied using a plasma spray technique. The ratio of HA to TCP in the HA/TCP coating is 65%/35%. At least 90% of HA material was in a crystalline phase^18^. Corundumized surface was also applied distal to the proximal coating. Characteristics of each porous coating are shown in Table 1. Cementless acetabular cups were used in all procedures, which consisted of the Continuum IT Shell (Zimmer-Biomet) in 90 hips and the Trabecular Metal Modula cup (Zimmer-Biomet) in 28 hips. Bearing materials consisted of metal-on-polyethylene for 72 hips and ceramic-on-polyethylene for 46 hips. In the TM group, metal head was used for 23 hips and ceramic head for 36 hips; in the VerSys group, metal head was used for 49 hips and ceramic head for 10 hips. Procedures were performed by experienced hip surgeons at 6 facilities according to the standard procedure and following the manufactures’ recommendations. The femoral canal was prepared as follows at all 6 centers; 1) a tapered awl was inserted to open the medullary canal and 2) the medullary canal was reamed until the desired canal size was achieved. There were no differences in femur preparations for stem insertion between the two groups. Postoperatively, immediate full weight-bearing with crutches was allowed.

**Table 1:** Characteristics of porous coatings.

	Porous tantalum	Fiber mesh
Geometry		
Material	Tantalum	Titanium
Pore size (μm)	400–600	100–400
Porosity (%)	75–85	40–50
Friction coefficient	0.98	0.63

Reprinted with permission from the ZimmerBiomet catalog.

**Figure 2:**
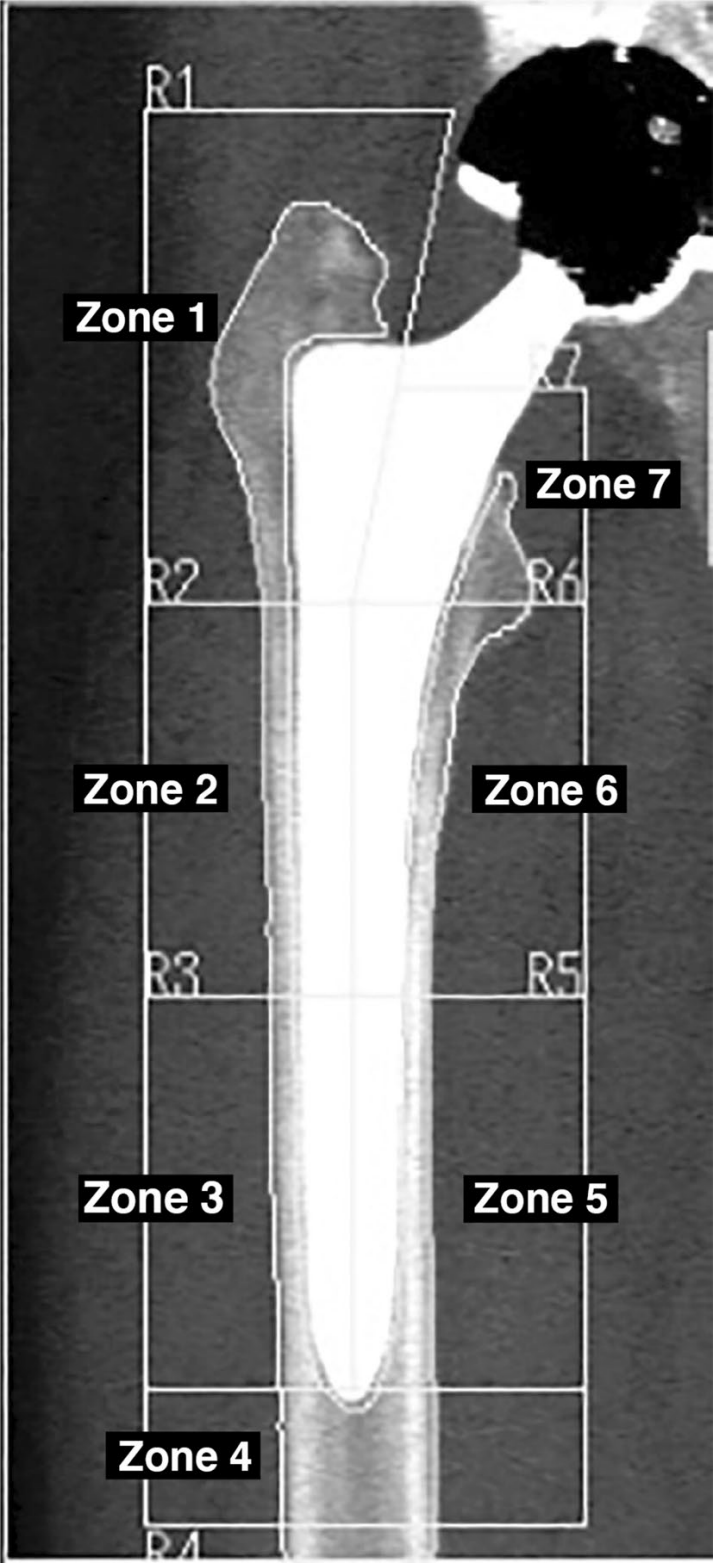
Seven reference zones Based on Gruen zones.

Periprosthetic BMD was measured within 1 week after surgery (baseline) and at 6, 12, and 24 months after surgery using 1 of 3 DEXA machines, Discovery (Hologic Inc., Marlborough, MA), QDR4500A (Hologic), or Lunar PRODIGY (GE Healthcare, Madison, WI). The relative change in BMD in each of the 7 Gruen zones^19^ (Fig. 2) was calculated by dividing the BMD from each examination by the baseline value. The ratio was expressed as a percentage of the baseline value. Patients were scanned in the supine position with foot positioning support to achieve reproducible internal hip rotation. All DEXA scans were performed by trained health professionals at each facility. Clinical outcome was assessed using the Japanese Orthopaedic Association (JOA) hip score^20^ before surgery and at 6, 12, and 24 months after surgery. The maximum possible JOA hip score is 100. It consists of four subcategories: pain (0–40 points), range of motion (0–20 points), ability to walk (0–20 points), and activities of daily living (0–20 points). The higher the score, the better the function of the hip joint. At each follow-up time point, all patients underwent radiological evaluation for the presence of radiolucency or osteolysis around the stem. Radiolucent lines were considered to be present if they were > 1.0 mm and occupied more than 50% of the interface in each Gruen zone.

### Statistics

BMD at Gruen zone 1 at 6 months after THA with VerSys HA-TCP Fiber Metal Taper stem was 88% compared with BMD at immediate post-operation as 100%^21^. When Trabecular Metal™Primary Hip Prosthesis is used for THA, assuming that BMD at 6 month after THA is 100%, 93 subjects are needed for each group with α = 0.05, β = 0.1 and S.D. = 25% to prove the superiority of Trabecular Metal™ Primary Hip Prosthesis to VerSys HA-TCP Fiber Metal Taper stem. Assuming 7 dropouts within 6 months after THA, the sample size of each group was initially determined as 100 subjects. Although we could not reach planned enrollment in 2 years period, we confirmed that a total sample size of 120 participants was needed to establish significance with a 22% difference between groups and a power of 0.8. Therefore, the enrollment had been stopped in September 2014 as originally planned. Age, body weight, height, and body mass index were compared between the two groups with the t-test. Gender was compared between the two groups with Fisher’s exact test. The relative change in BMD and JOA hip scores at 6, 12, and 24 months were compared between the two groups with the Wilcoxon rank sum test. Baseline BMD values and the paired values at each follow-up time point in each group were compared with the Wilcoxon signed rank test. Statistical analysis was performed using the JMP 15.1.0 software package (SAS Institute, Cary, NC); p < 0.05 was considered significant.

## Results

**Figure 3:**
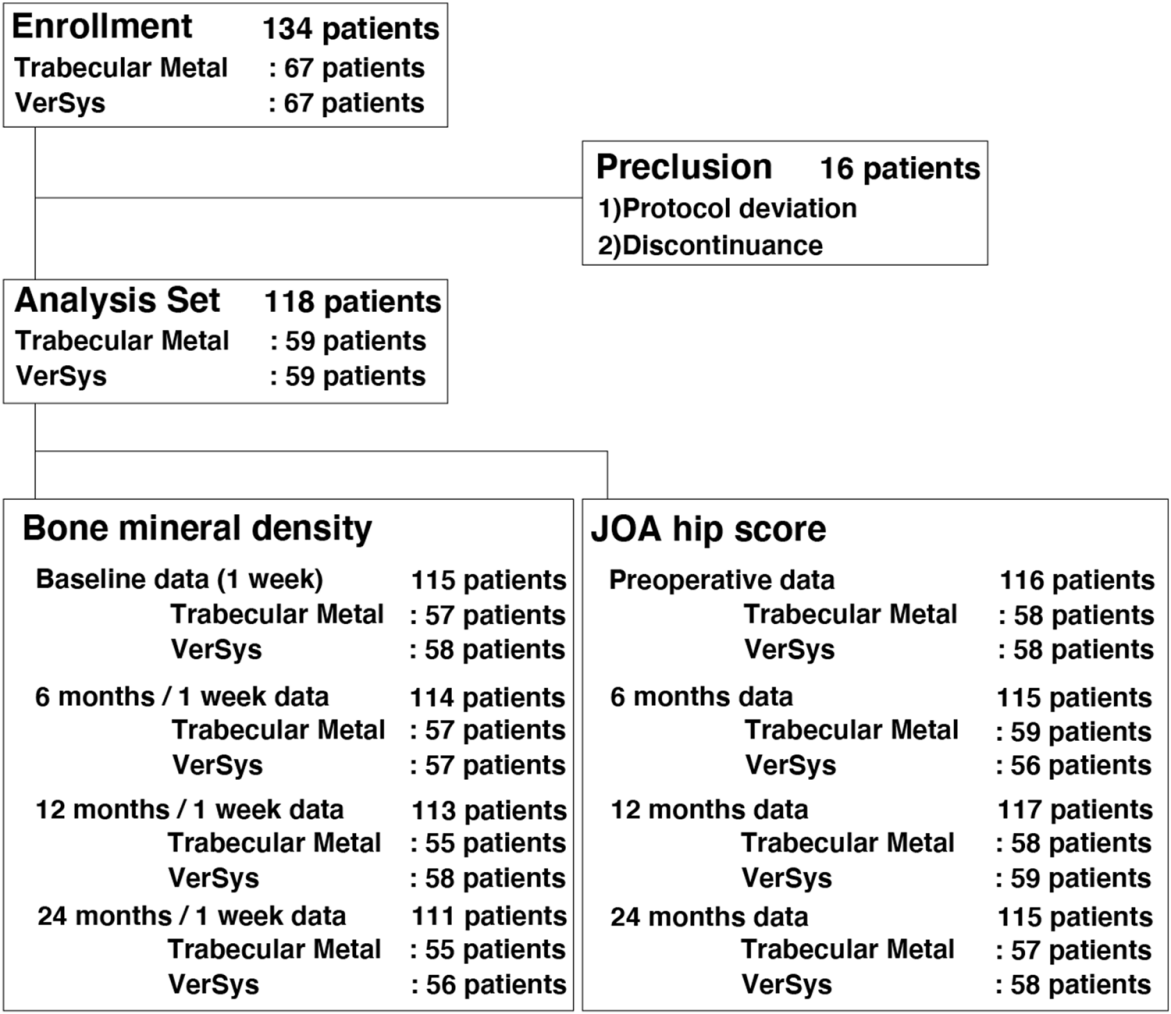
Flowchart.

Of 134 patients assessed for eligibility, 16 patients were excluded due to protocol deviation (including violation of inclusion or exclusion criteria) and discontinuation as a result of greater trochanteric fracture as a complication (Fig. 3). A total of 118 patients were included in the analysis (mean age: 61.5 years, 107 females and 11 males) (Fig. 3). There were no significant differences in gender, age, body weight, height, or body mass index between the two groups (Table 2). Of the 118 patients, 1 developed a postoperative wound infection, which healed with conservative treatment. There were no other complications. No revision surgeries were performed during the study period.

**Table 2:** Preoperative characteristics.

	Trabecular metal (n = 59)	VerSys (n = 59)	*P* value
Gender (female/male)	52/7	55/4	0.53
Age (years)*	62.1 ± 8.5	60.9 ± 8.0	0.43
Body weight (kg)*	57.8 ± 11.6	55.1 ± 9.0	0.18
Height (cm)*	153.1 ± 6.5	153.6 ± 6.1	0.64
Body mass index (kg/m^2^)*	24.5 ± 4.0	23.4 ± 3.5	0.09

*Values are given as means ± standard deviation.

### Primary outcome

**Figure 4:**
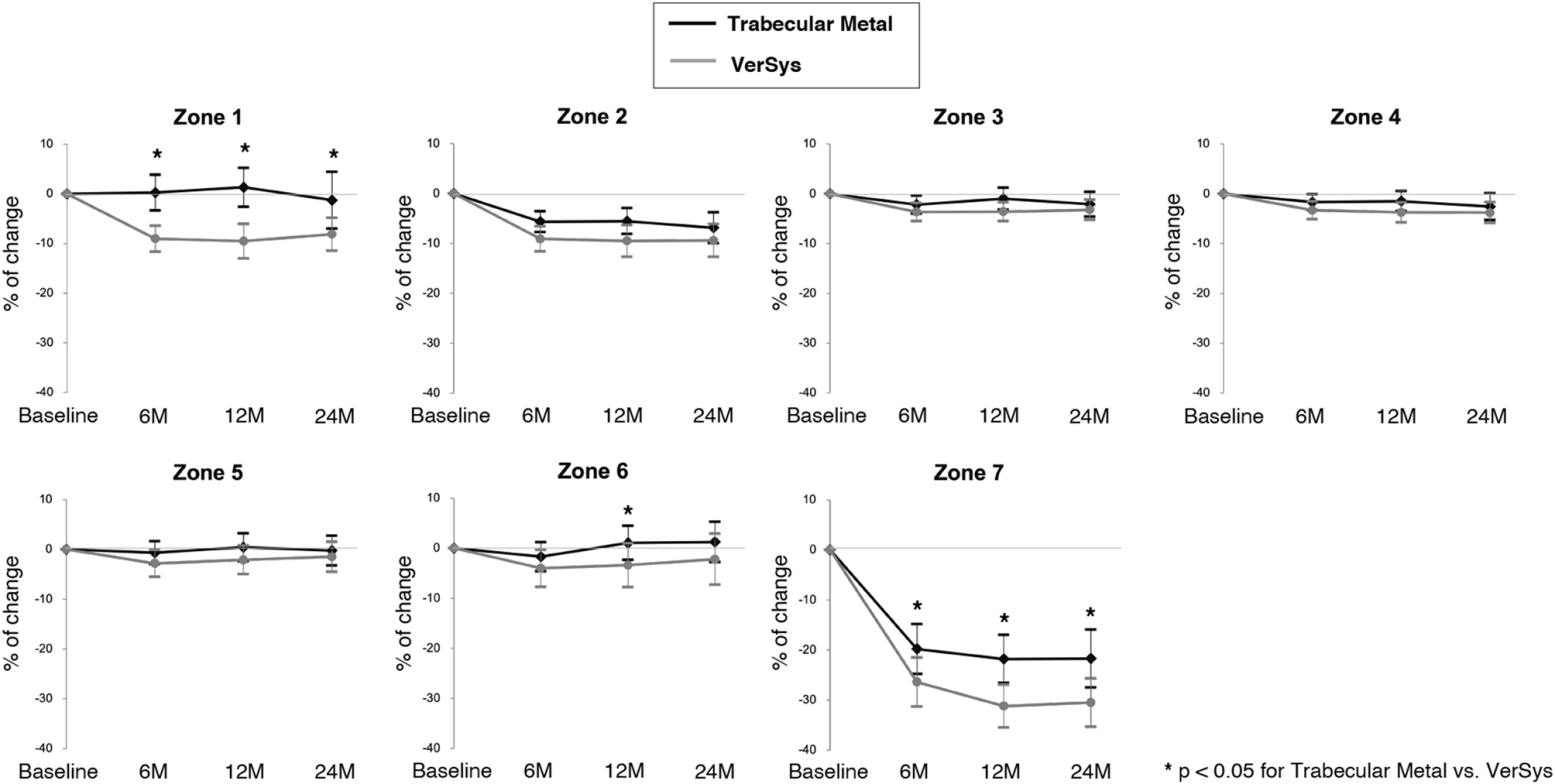
Relative change in bone mineral density in each Gruen zone. In the proximal periprosthetic region (zones 1 and 7), the Trabecular Metal group had a significantly smaller reductions in bone mineral density than the VerSys group throughout the study period.

In Gruen zone 1, the VerSys group had a mean BMD decrease of 9.5% from baseline through 12 months after surgery, while the Trabecular Metal group had no mean decrease in BMD from baseline through 12 months after surgery (Fig. 4). At each follow-up time point, there was a significant difference in the relative change in BMD between the two groups (p < 0.0001 at 6 months, p < 0.0001 at 12 months, and p = 0.003 at 24 months, respectively) (Fig. 4). Gruen zone 7 had the greatest mean BMD decrease in both groups, while the Trabecluar Metal group has a significantly smaller relative change in BMD than the VerSys group at all follow-up time points (p < 0.035 at 6 months, p < 0.003 at 12 months, and p = 0.029 at 24 months, respectively) (Fig. 4).

In the VerSys group, there were significant reductions in BMD compared to baseline at each measurement point in all zones, except for zone 6 at 24 months. In the Trabecular Metal group, there were no significant reductions in BMD compared to baseline in zones 1, 5, and 6 throughout the study period (Table 3).

**Table 3:** Bone mineral density at each time point relative to baseline by zone and stem type.

	Zone 1		Zone 2		Zone 3		Zone 4	
	BMD (g/cm^2^)*	p	BMD (g/cm^2^)*	p	BMD (g/cm^2^)*	p	BMD (g/cm^2^)*	p
**TM**								
Baseline	0.63 (0.36–1.11)		1.39 (0.89–2.25)		1.64 (1.00–2.37)		1.61 (1.01–2.40)	
6 months	0.64 (0.33–1.17)	0.55	1.34 (0.73–2.35)	< .0001	1.63 (1.01–2.48)	0.0031	1.54 (1.01–2.44)	0.0042
12 months	0.62 (0.35–1.20)	0.72	1.34 (0.72–2.24)	< .0001	1.63 (1.04–2.33)	0.13	1.57 (0.98–2.36)	0.015
24 months	0.62 (0.14–1.35)	0.58	1.34 (0.58–2.30)	< .0001	1.62 (1.02–2.15)	0.041	1.55 (0.77–2.35)	0.015
**VerSys**								
Baseline	0.65 (0.27–1.09)		1.46 (0.88–2.17)		1.59 (1.08–2.36)		1.55 (0.95–2.32)	
6 months	0.60 (0.25–1.04)	< .0001	1.35 (0.73–1.93)	< .0001	1.56 (0.95–2.49)	< .0001	1.51 (1.00–2.18)	< .0001
12 months	0.59 (0.28–0.94)	< .0001	1.36 (0.68–1.88)	< .0001	1.60 (0.99–2.33)	< .0001	1.50 (1.00–2.19)	0.0001
24 months	0.60 (0.29–0.97)	< .0001	1.38 (0.64–1.98)	< .0001	1.62 (1.02–2.31)	0.0009	1.50 (0.98–2.16)	0.0001

	Zone 5		Zone 6		Zone 7			
	BMD (g/cm^2^)*	p	BMD (g/cm^2^)*	p	BMD (g/cm^2^)*	p		
**TM**								
Baseline	1.67 (0.95–2.58)		1.29 (0.84–1.94)		0.95 (0.28–1.72)			
6 months	1.67 (1.04–2.72)	0.13	1.31 (0.66–2.01)	0.14	0.74 (0.29–1.84)	< .0001		
12 months	1.72 (1.14–2.57)	0.35	1.42 (0.55–2.09)	0.76	0.74 (0.25–1.76)	< .0001		
24 months	1.65 (0.97–2.51)	0.38	1.36 (0.71–2.11)	0.69	0.69 (0.27–1.80)	< .0001		
**VerSys**								
Baseline	1.69 (0.84–2.50)		1.32 (0.85–1.87)					
6 months	1.61 (0.94–2.58)	0.0041	1.34 (0.58–1.93)	0.019	0.99 (0.43–1.71)	< .0001		
12 months	1.59 (1.10–2.29)	0.0093	1.34 (0.58–1.89)	0.024	0.71 (0.26–1.47)	< .0001		
24 months	1.62 (0.98–2.19)	0.022	1.33 (0.49–2.19)	0.18	0.67 (0.23–1.22)	< .0001		

*BMD* bone mineral density, *TM* Trabecular Metal.

*Values are given as the medians (range).

### Secondary outcome

Both groups demonstrated similar improvement in JOA hip scores over the study period (Table 4). Radiologically, radiolucent line was observed in 7 patients at 24 months after surgery, 2 in the Trabecular Metal group and 5 in the VerSys group. In 6 of 7 patients, the radiolucent line was found only in zone 3. One patient in the VerSys group had radiolucent lines in Gruen zones 3, 4, 5, and 6, but no evidence of stem loosening was observed. There was no osteolysis around the stems at 24 months after surgery.

**Table 4:** Japanese Orthopaedic Association hip score by group.

Score	Evaluation period		Trabecular Metal	VerSys	*p *value
Pain	Before surgery		15.0 ± 7.6	14.5 ± 8.8	0.74
	After surgery	6 months	37.2 ± 3.5	37.1 ± 4.0	0.93
		12 months	37.4 ± 4.0	36.6 ± 6.1	0.40
		24 months	37.2 ± 4.3	38.3 ± 2.6	0.11
ROM	Before surgery		11.7 ± 4.1	11.3 ± 4.2	0.61
	After surgery	6 months	16.6 ± 2.8	17.1 ± 2.8	0.34
		12 months	17.3 ± 2.4	17.3 ± 2.5	0.96
		24 months	17.3 ± 2.5	17.3 ± 2.4	0.88
Walk	Before surgery		10.6 ± 3.0	10.6 ± 3.2	0.97
	After surgery	6 months	16.6 ± 4.6	16.9 ± 3.5	0.66
		12 months	18.1 ± 3.4	17.9 ± 2.8	0.79
		24 months	18.2 ± 3.0	18.1 ± 2.9	0.82
ADL	Before surgery		13.1 ± 2.4	12.5 ± 1.8	0.18
	After surgery	6 months	17.4 ± 2.8	16.9 ± 3.1	0.37
		12 months	17.6 ± 2.8	17.6 ± 2.7	0.90
		24 months	17.9 ± 2.7	18.4 ± 2.1	0.28
Total	Before surgery		50.3 ± 10.6	48.3 ± 11.2	0.30
	After surgery	6 months	87.7 ± 8.8	88.0 ± 8.5	0.87
		12 months	90.4 ± 7.8	89.1 ± 10.6	0.43
		24 months	90.2 ± 8.3	90.8 ± 9.1	0.71

Score values are given as means ± standard deviation.

*ROM* range of motion, *ADL* activities of daily living.

## Discussion

Previous clinical studies have examined several factors that influence periprosthetic bone remodeling, including stem design^22–24^, material^25^, and extent of porous coating^26,27^. However, no clinical studies have focused on the effect of porous surface characteristics on periprosthetic bone remodeling. In this study, we examined the effects of porous tantalum on the periprosthetic bone remodeling around the femoral stem. We found that a proximally coated stem with porous tantalum was superior to a conventional stem with titanium fiber mesh in terms of bone remodeling.

Regarding the bone ingrowth with porous tantalum, an experimental study demonstrated mean ingrowth of 41.5% at 4 weeks after implant insertion, resulting in a mean shear fixation strength of at least 18.5 MPa^28^, which was substantially higher than with other porous materials that have less volumetric porosity^29^. By contrast, another experimental study showed that mean bone ingrowth with a fiber metal implant that had a calcium phosphate coating was 16.5%, with a mean shear strength of 2.75 MPa^30^. Based on the results of these experimental studies, the proximally coated stem with porous tantalum used in this study might provide earlier and stronger proximal fixation than a conventional stem with titanium fiber mesh, which may lead to differences in periprosthetic bone remodeling between the two stem types.

Two studies^24,31^ assessing periprosthetic bone remodeling around the proximally coated stem with titanium fiber mesh used in this study have shown significant bone loss, especially in the proximal periprosthetic region (Gruen zones 1 and 7). In those studies, mean BMD loss at 1 year after surgery was approximately 20–30% in zone 1 and approximately 30% in zone 7^24,31^, which were similar to the findings of this study. Yamauchi et al. pointed out the possibility that stem fixation in the central area of the stem’s corundumized surface may contribute to more distal fixation^32^. Thus, the central corundumized surface might contribute to the substantial bone loss in the proximal periprosthetic region.

Tanzer et al. performed a prospective randomized trial to assess the effect of a calcium phosphate coating on femoral bone remodeling after cementless THA using proximally porous-coated stems with titanium fiber mesh^33^. They demonstrated that HA-TCP coated stems had significantly less femoral bone loss than uncoated stems at 2-year follow-up^33^. They thought that the increased retention of BMD with the HA-TCP-coated stems most likely resulted from increased bone formation in the periprosthetic regions secondary to the coating, increased load transfer between the implant and bone because of enhanced osseointegration, or both^33^. Although this study could not assess the pure effect of a calcium phosphate coating on periprosthetic bone remodeling due to the different porous coatings between the two stems, the results of this study do not refute the possibility that a calcium phosphate coating contributes to less periprosthetic bone loss.

However, we found no differences in the clinical outcomes between the two groups. Previous clinical studies reported similar clinical results between stems, despite differences in periprosthetic bone remodeling^33–35^. Taken together, this suggests that periprosthetic bone loss does not affect short-term clinical outcomes. Regarding long-term effects, the clinical relevance of periprosthetic bone loss remains unclear. Several studies have reported that periprosthetic bone loss has no clinical impact based on long-term results^36,37^, but other studies have shown continuous bone loss in the proximal region over a period of more than 10 years^38,39^. Furthermore, periprosthetic fractures have been reported to be the most important cause of long-term failure after cementless THA, with a cumulative probability of 13% after 29 years^40^. Thus, we believe that it is worthwhile to reduce the amount of periprosthetic bone loss.

This study has several limitations. First, since the two stems differed in aspects besides the type of porous coating, the present study could not attribute the effect on prosthetic bone remodeling to differences in porous coating type alone. However, we were able to evaluate the effect of porous tantalum on bone remodeling in this randomized controlled trial. Second, due to the multicenter nature of this study, periprosthetic BMD was measured using 3 different DEXA machines. However, in this study, we assessed the change in BMD from baseline. We believe that the effect of the different DAXA machines is small because each patient is his or her own control. Third, the observation period of 2 years only demonstrated early trends. Further study is needed to clarify the long-term effect of porous tantalum on periprosthetic bone remodeling.

In conclusion, this study demonstrated that a proximally coated stem with porous tantalum was superior to a conventional stem with titanium fiber mesh in terms of periprosthetic bone remodeling.

## Data Availability

The datasets generated during and/or analyzed during the current study are available from the corresponding author on reasonable request.
